# Analysis of Gut Characteristics and Microbiota Changes with Maternal Supplementation in a Neural Tube Defect Mouse Model

**DOI:** 10.3390/nu15234944

**Published:** 2023-11-28

**Authors:** Juan Antonio Cordero-Varela, Marta Reyes-Corral, Miguel Lao-Pérez, Beatriz Fernández-Santos, Fernando Montenegro-Elvira, Lluis Sempere, Patricia Ybot-González

**Affiliations:** 1 Institute of Biomedicine of Seville (IBiS)/Virgen del Rocío University Hospital/CSIC/University of Seville, 41013 Seville, Spain; jacordero-ibis@us.es (J.A.C.-V.); mlao-ibis@us.es (M.L.-P.); beatrizmfersan@gmail.com (B.F.-S.); fernandomontelv@gmail.com (F.M.-E.); luissempere@yahoo.es (L.S.); 2 Consejo Superior de Investigaciones Científicas (CSIC), Spain

**Keywords:** neural tube defects, microbiota, metagenomics, pregnancy, prevention, folic acid, inositol, *loop-tail*, Wnt/PCP

## Abstract

Adequate nutrient supply is crucial for the proper development of the embryo. Although nutrient supply is determined by maternal diet, the gut microbiota also influences nutrient availability. While currently there is no cure for neural tube defects (NTDs), their prevention is largely amenable to maternal folic acid and inositol supplementation. The gut microbiota also contributes to the production of these nutrients, which are absorbed by the host, but its role in this context remains largely unexplored. In this study, we performed a functional and morphological analysis of the intestinal tract of *loop-tail* mice (*Vangl2* mutants), a mouse model of folate/inositol-resistant NTDs. In addition, we investigated the changes in gut microbiota using 16S rRNA gene sequencing regarding (1) the host genotype; (2) the sample source for metagenomics analysis; (3) the pregnancy status in the gestational window of neural tube closure; (4) folic acid and (5) D-*chiro*-inositol supplementation. We observed that *Vangl2^+/Lp^* mice showed no apparent changes in gastrointestinal transit time or fecal output, yet exhibited increased intestinal length and cecal weight and gut dysbiosis. Moreover, our results showed that the mice supplemented with folic acid and D-*chiro*-inositol had significant changes in their microbiota composition, which are changes that could have implications for nutrient absorption.

## 1. Introduction

During pregnancy, there is a high demand for vitamins and nutrients necessary for biological processes involved in the development of fetal and maternal tissues [1]. To ensure a healthy pregnancy, the World Health Organization (WHO) recommends that pregnant women should exercise regularly and follow a healthy diet, including a folic acid (FA) supplement of 0.4 mg per day [2]. FA is involved in various essential metabolic functions such as DNA repair, replication and methylation, and synthesis of nucleotides, vitamins and certain amino acids. The benefit of FA supplementation during the periconceptional period has been demonstrated to reduce the risk of congenital diseases such as neural tube defects (NTDs) [3,4,5], congenital heart disease [6,7] and craniofacial malformations [8,9,10].

NTDs are the second most common structural malformation in humans, with a prevalence that—depending on the geographic location—varies from 1/3000 pregnancies in the US to 1/1000 in Europe and the Middle East and 3/1000 in northern China [11]. NTDs appear in the early stages of embryogenesis, during the course of neurulation, a process that in humans occurs during the fourth week of gestation and involves the folding of the neural plate and dorsal fusion to form the neural tube, which is the precursor of the central nervous system [12]. Failure to complete the closure process in the posterior region of the neural tube causes myelomeningocele or spina bifida [13], whereas incomplete cranial closure causes anencephaly [14] and failed closure along the entire spinal neural tube and part of the cranial neural tube develops craniorachischisis. Anencephaly and craniorachischisis are incompatible with life, while most patients with spina bifida can survive to adulthood, although they are likely to suffer severe lifelong disabilities [12]. Periconceptional intake of FA has been shown to greatly reduce the incidence of NTDs, preventing 49–71% of cases; indeed, FA fortification of a variety of cereal products became mandatory in countries such as Canada and the US [15,16]. The remaining cases of NTDs are considered folate-resistant, and in some cases, compounds such as *myo*- and D-*chiro*-inositol have been shown to be alternatives for the prevention of NTDs in both mice and humans [17,18,19]. For instance, in the folate-resistant NTD *curly-tail* mouse model, D-*chiro*-inositol was more effective than its isomer *myo*-inositol and reduced the frequency of spina bifida by 73−86%, whereas *myo*-inositol caused a reduction of 53−56%. These findings suggest that both D-*chiro*-inositol and *myo*-inositol have a protective effect against folate-resistant NTDs in mice, although the specific mechanism by which inositol normalizes neural tube closure is not fully understood. It has been suggested that inositol may correct a cell proliferation defect that contributes to the development of NTDs [17,18].

Like other vitamins, folate (vitamin B9) is not synthesized by mammalian cells and needs to be obtained through intestinal absorption from external sources, such as food and the gut microbiome [20]. Folate is naturally present in foods such as green leafy vegetables, fruits, cereals and liver products, and is mainly absorbed in the small intestine. Besides dietary sources, intestinal bacteria in the colon are also significant contributors to folate, and the presence of folate transporters in the human colon suggests that bacterially biosynthesized folate may be involved in host metabolism [21]. According to a study in piglets, bacterial biosynthesis may provide at least 18% of the dietary folate requirement [22]. In addition, B vitamin intake influences the composition of the maternal gut microbiota during pregnancy, which may modulate nutrient acquisition and absorption [23]. There is substantial literature dedicated to exploring the role of folic acid in preventing NTDs (reviewed in [24]) and how dietary folic acid variations may influence gut microbiota (reviewed in [25]). However, there has been limited attention given to understanding the potential role that the microbiota associated with the supplemented diet may be playing in disease prevention.

Our research is aimed at understanding the etiology of NTDs, which may shed light on why some of these defects are resistant to maternal dietary supplementation with different nutrients. *Loop-tail* mice are mutants for the *Vangl2* gene, a core member of the non-canonical Wnt/PCP pathway that is involved in the formation of the neural tube through the regulation of cytoskeletal dynamics, determining cell movement and tissue arrangement [26,27]. We have previously shown that *loop-tail* mice develop folic acid- and inositol-resistant NTDs in homozygosity and heterozygosity [28,29]. Additionally, a study in *Xenopus* showed that Vangl2 coordinates cell rearrangements during gut elongation and that mutations in *Vangl2* are associated with alterations in the epithelial morphogenesis of the gut [30]. Given the association of *Vangl2* mutations with the development of folic acid- and inositol-resistant NTDs [28,29] and with alterations in gut morphogenesis [30], in the present study we aimed to explore the intestinal tract of *Vangl2^+/Lp^* mice. First, we studied the macroscopic and functional characteristics of the intestinal tract of *loop-tail* mice, which revealed that *Vangl2* mutation alters the intestinal length and cecal weight but not the gut transit time or stool production. Second, we performed a descriptive metagenomics analysis of the gut microbiota of *Vangl2^+/Lp^* female mice fed a control diet or supplemented with folic acid and D-*chiro*-inositol during pregnancy. Our results showed that *Vangl2* mutation is associated with certain gut dysbiosis and that these dietary supplements—commonly used for the prevention of NTDs—can alter the microbiome composition, which may affect the absorption of nutrients.

## 2. Materials and Methods

### 2.1. Mice, Sampling and Experimental Setup

The *loop-tail* (*Vangl2^Lp^*) inbred strain carrying the *Vangl2* mutation was originally obtained from Jackson Laboratories (Bar Harbor, ME, USA), and it was maintained in a C3H background. These mice have a mutation in the *Vangl2* gene, a core member of the non-canonical Wnt/PCP pathway involved in the closure of the neural tube [26,27]. Mice were kept in a 12 h light/dark cycle, with *ad libitum* access to water and rodent chow (Teklad Global Rodent Diet 2014S, Envigo; Italy)*. Vangl2^+/Lp^* females were paired with *Vangl2^+/Lp^* male mice and the presence of the vaginal mucus plug was inspected at day E0.5. Two weeks prior to pairing, the food for the folic acid (FA)-supplemented mice (both males and females) was changed to one containing 10 ppm of FA, five times more than the regular food. The D-*chiro*-inositol (CI; Santa Cruz Biotechnology, sc-221469; Dallas, TX, USA) was administered to the CI-supplemented group from day E1.5 to E11.5, and was diluted in water at a concentration of 800 µg per gram of mouse weight per day [18]. On day E12.5, fresh feces were collected and stored at −80 °C, the mice were sacrificed, and their cecum were extracted, frozen in liquid nitrogen and stored at −80 °C until further use.

**Figure 1:**
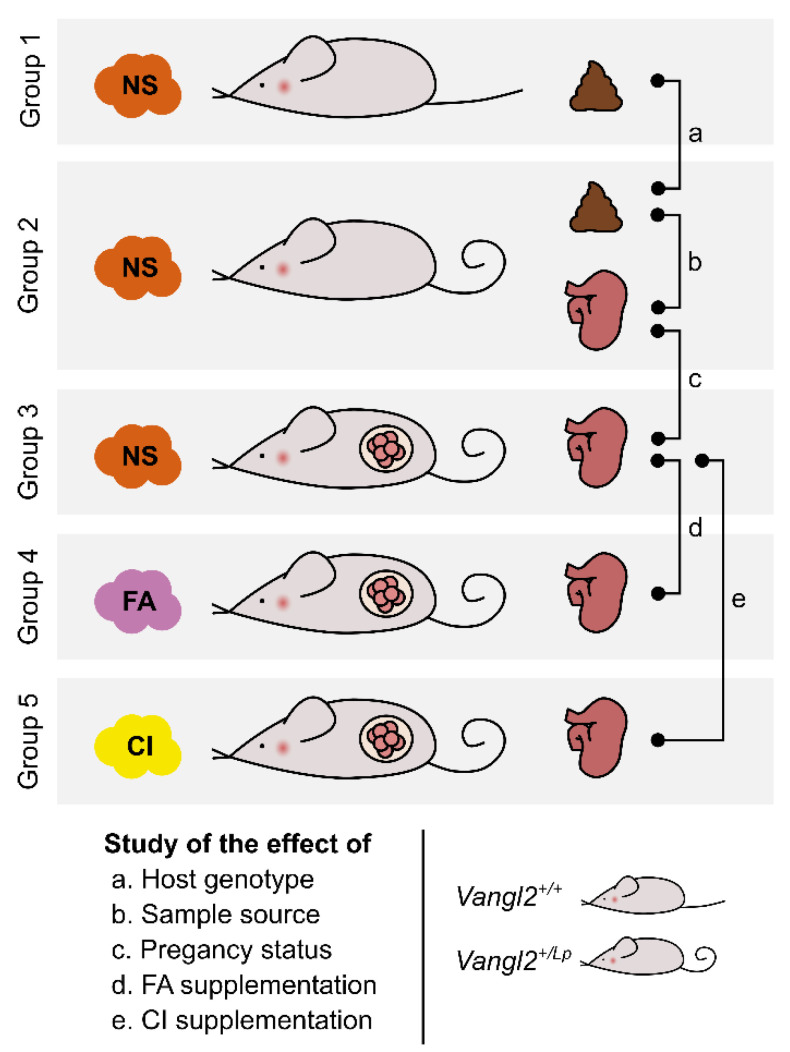
Experimental design for the study of gut microbiota in *loop-tail* mice. We investigated the changes in gut microbiota in relation to the host genotype (*Vangl2^+/+^* vs *Vangl2^+/Lp^*), the sample source (feces vs cecum), the pregnancy status, and folic acid (FA) or D-*chiro*-inositol (CI) supplementation. To this end, feces and/or cecum samples were collected from five different groups of *n* = 9 female mice each: wild type *Vangl2^+/+^* non-supplemented (NS) and non-pregnant mice (group 1); heterozygous *Vangl2^+/Lp^* non-supplemented and non-pregnant mice (group 2); and heterozygous *Vangl2^+/Lp^* pregnant mice non-supplemented (group 3); supplemented with FA (group 4); and supplemented with CI (group 5).

Female mice were randomly allocated into five different groups of *n* = 9 mice each (Figure 1). Group 1: wild type *Vangl2^+/+^* non-supplemented and non-pregnant mice, from which feces were collected (9 fecal samples). Group 2: heterozygous *Vangl2^+/Lp^* non-supplemented and non-pregnant mice, from which both feces and cecum were collected (18 samples: 9 fecal and 9 cecal). Group 3: heterozygous *Vangl2^+/Lp^* pregnant mice non-supplemented from which cecum were collected (9 cecal samples). Group 4: heterozygous *Vangl2^+/Lp^* pregnant mice FA-supplemented from which cecum were collected (9 cecal samples). Group 5: heterozygous *Vangl2^+/Lp^* pregnant mice CI-supplemented from which cecum were collected (9 cecal samples). In total, 54 samples were collected (18 fecal samples and 36 ceca) and used for 16S rRNA gene sequencing to analyze the gut microbiota.

All procedures involving experimental animals were performed in compliance with local, national and European animal welfare laws, guidelines and policies.

### 2.2. Functional and Morphological Analysis of the Intestinal Tract of Vangl2^+/+^ and Vangl2^+/Lp^ Mice

For the functional analysis of the intestinal tract, total gastrointestinal transit time and stool production were measured as previously described [31,32]. *Vangl2^+/+^* and *Vangl2^+/Lp^* age-matched female mice (13 ± 3 weeks old; *n* = 10 per group) were placed in individual cages devoid of bedding and, after fasting for 1 h, 0.3 mL of blue food coloring dye (Dr. Oetker) dissolved in distilled water was administered to each mouse by oral gavage. Total gastrointestinal transit time was calculated from the time of administration to the first visualization of dye in the stool. Maximum observation time was 3 h and a blue-colored stool was observed within this timeframe in all animals analyzed. Stool output in 1 h and average stool weight were measured by placing individual animals in separate cages and collecting all fecal pellets produced over a 1 h period (10 to 11 a.m.). Stools were desiccated overnight at 75 °C and stool water content was calculated as the difference between wet and dry weight expressed as a percentage.

For the morphological analysis of the intestinal tract of *Vangl2^+/+^* and *Vangl2^+/Lp^* mice, the intestines were macroscopically assessed as described by others [33]. The animals were sacrificed by cervical dislocation, followed by complete removal of the intestine from the stomach to the anus. The length of the small intestine and colon was measured, as well as the weight of the cecum and its contents. The length of the whole intestine was calculated as the sum of the lengths of the small intestine and the colon. For comparison, the data collected were normalized to the weight of each animal.

### 2.3. DNA Extraction and 16S rRNA Gene Sequencing

DNA from the 54 fecal and cecal samples was extracted with the EZNA Stool DNA Kit (Omega Bio-Tek, GA, USA), following the manufacturer’s protocol. All samples were diluted to 5 ng/mL before sequencing. The primers used were the Illumina 341F and 805R, targeting the V3–V4 region of the 16S rRNA gene, including the adapters for sequencing (forward, 5′-TCGTCGGCAGCGTCAGATGTGTATAAGAGACAGCCTACGGGNGGC-WGCAG; reverse, 5′-GTCTCGTGGGCTCGGAGATGTGTATAAGAGACAGGACTACHVGGGTATCTAATCC). The sequencing was performed using Illumina MiSeq at the Genomics Core Facility of the Institute of Biomedicine of Seville.

### 2.4. Analysis of 16S rRNA Sequencing Data

Amplicon libraries consisting of demultiplexed, 300 nucleotide-long, paired-end FASTQ files were processed with Mothur v1.44.11 [34]. Quality control was assessed by filtering only sequences between 440 and 470 nucleotides without ambiguous calls, as well as considering clustering sequences with four or fewer different nucleotides as identical sequences. SILVA v138 [35] and Greengenes gg_13_8_99 [36] databases were employed to align and classify sequences, respectively. OTU counts table, taxonomy, rooted phylogenetic tree and metadata files were imported to R v4.2.2 (R Core Team, 2022) via the phyloseq package v1.42.0 [37]. Samples were rarefied to the smallest OTU count (38,687) to compute diversity metrics. Taxa not present (zero counts) in at least nine samples (smallest sample size of the treatment groups) were removed. Instead of using OTUs directly, all taxa were summarized at the genus level.

In recent years, there has been an increasing consensus that alpha diversity is best described by the Hill numbers rather than by classical diversity measures [38]. For this reason, we calculated Hill’s Diversity (qD) for each group, and for different numbers (q): 0 (equals to species richness), 1 (modified version of Shannon index) and 2 (equivalent to Simpson index). The higher the number, the stronger focus on dominant taxa. A Mann–Whitney test was applied to alpha diversity values to determine differences among groups of interest.

Beta diversity was explored through a non-metric multidimensional scaling (NMDS) using the ecodist package v2.0.9 [39]. Since Euclidean distance displayed the lowest stress value, we chose this metric for follow-up calculations. Global differences in microbial composition were tested using a permutational ANOVA (Permanova) using the vegan package v2.6-4 [40] while a pairwise permanova was performed to discover differences between groups of interest. A homogeneity of molecular variance (HOMOVA) test was also applied to confirm significant differences were not due to differences in dispersion. Differential abundance analysis of taxa was performed with a Zero-inflated Gaussian mixture model using the metagenomeSeq method [41] with microbiomeMarker package v1.6.0 [42].

### 2.5. Statistical Analysis

**Figure 2:**
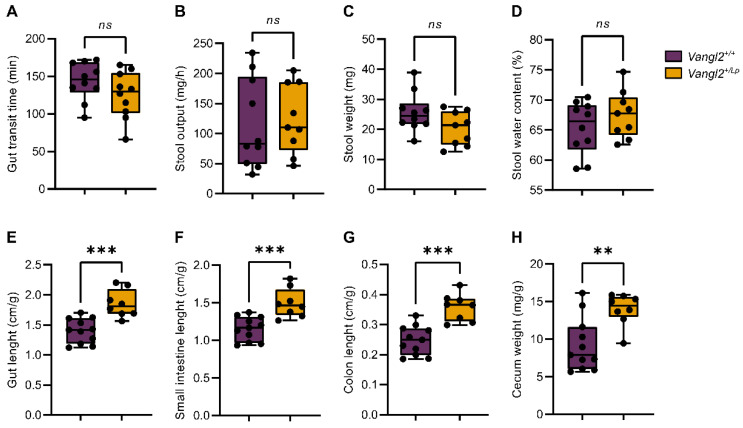
Functional and macroscopic morphological analysis of the intestinal tract of *Vangl2^+/+^* (purple) and *Vangl2^+/Lp^* (dark yellow) female mice. (**A**) Total gastrointestinal transit time measured after oral gavage of dye. (**B**) Stool production in 1 h. (**C**) Average stool weight. (**D**) Stool water content. (**E**) Total gut length. (**F**) Small intestine length. (**G**) Colon length. (**H**) Cecum weight. Measurements in (**E**–**H**) were normalized to the weight of each animal (in grams). Unpaired two-sided *t*-test was applied to study differences between *Vangl2^+/+^* and *Vangl2^+/Lp^* mice: *ns*, non-significant; ** *p* < 0.01; *** *p* < 0.001 (*n* = 8–10 mice per group).

**Figure 3:**
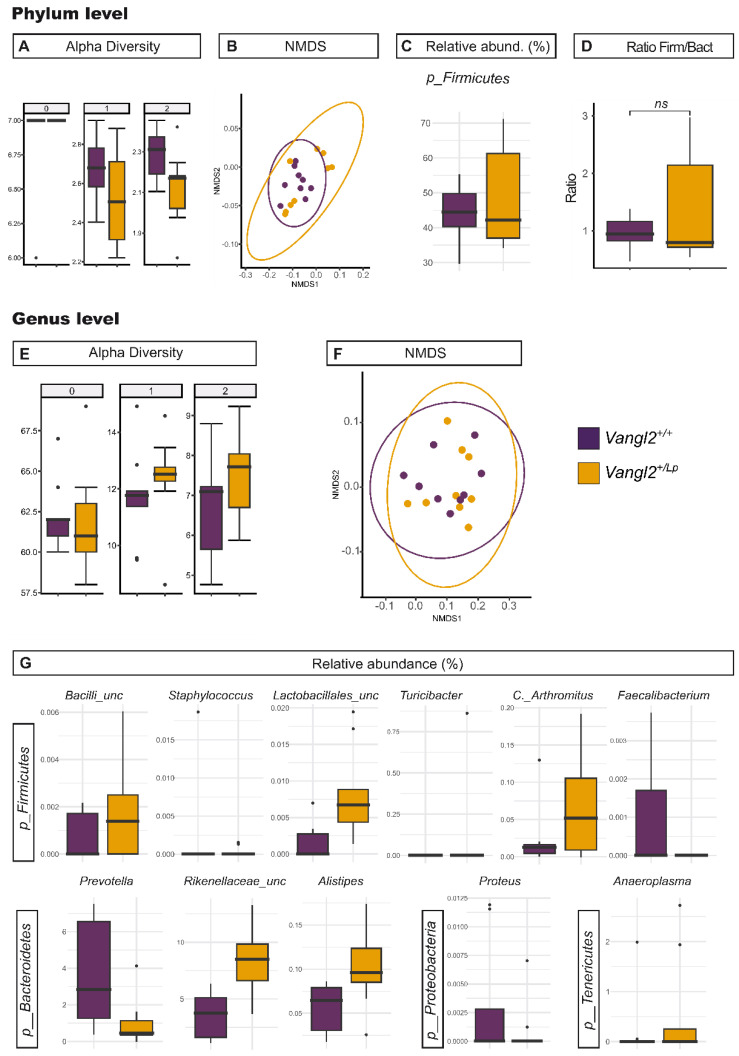
Effect of the host genotype on gut microbiota. Richness and diversity measures of the microbiota from *Vangl2^+/+^* (purple) and *Vangl2^+/Lp^* (dark yellow) female mice stool samples, according to the phylum (**A**–**D**) and the genus (**E**–**G**). (**A**,**E**) Alpha diversity described by the Hill numbers for each group: 0, species richness; 1, modified version of Shannon index; 2, equivalent to Simpson index. The higher the number, the stronger focus on dominant taxa. A Mann–Whitney test was applied to alpha diversity values to determine differences among groups of interest. (**B**,**F**) Non-metric multidimensional scaling (NMDS) analysis for beta microbiome diversity. (**C**,**G**) Significant difference (*p* < 0.05) between *Vangl2^+/+^* and *Vangl2^+/Lp^* female mice stool samples at phylum and genera level. (**D**) *Firmicutes/Bacteroidetes* ratio.

**Figure 4:**
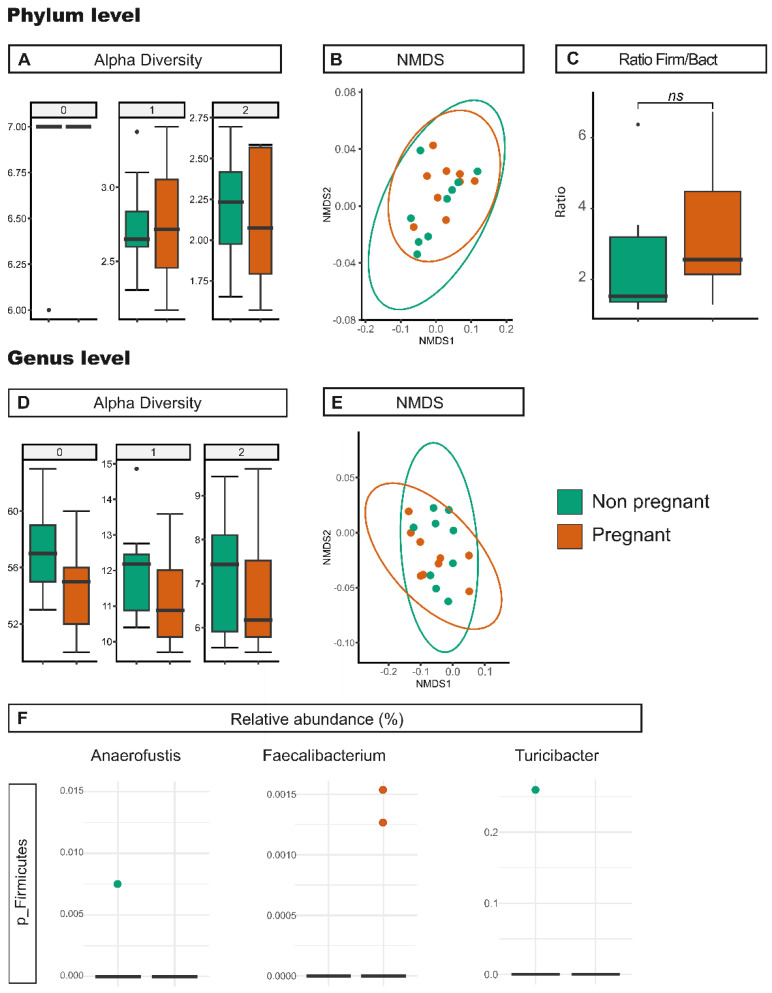
Effect of the pregnancy status on gut microbiota. Richness and diversity measures of the microbiota from *Vangl2^+/Lp^* non-pregnant (green) and *Vangl2^+/Lp^* pregnant (orange) female mice cecum samples, according to the phylum (**A**,**B**) and the genera (**C**–**E**). (**A**,**D**) Alpha diversity described by the Hill numbers for each group: 0, species richness; 1, modified version of Shannon index; 2, equivalent to Simpson index. Higher number means a stronger focus on dominant taxa. A Mann–Whitney test was applied to alpha diversity values to determine differences among groups of interest. (**B**,**E**) NMDS analysis for beta microbiome diversity. (**C**) *Firmicutes/Bacteroidetes* ratio. (**F**) Significant differences (*p* < 0.05) in the relative abundance between *Vangl2^+/Lp^* non-pregnant and *Vangl2^+/Lp^* pregnant female mice cecum samples at genera level.

**Figure 5:**
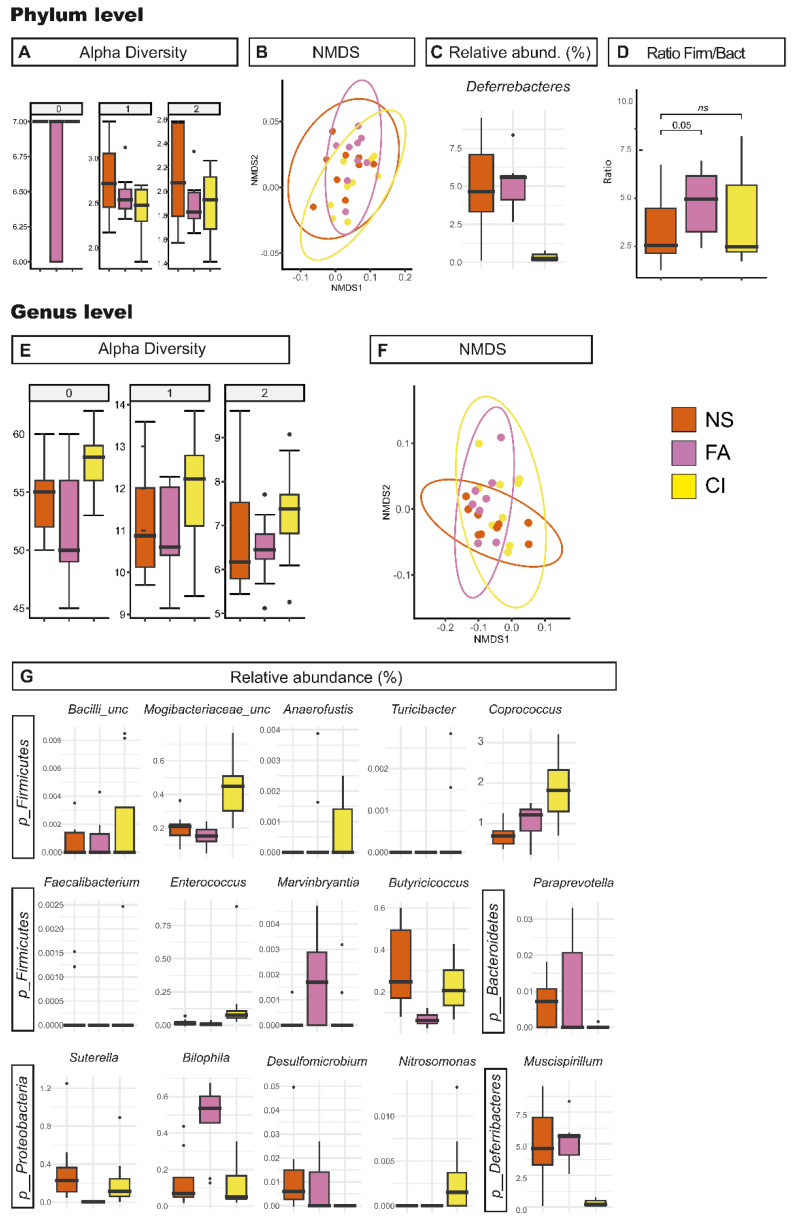
Effect of the dietary supplementation on gut microbiota. Richness and diversity measures of the microbiota from *Vangl2^+/Lp^* pregnant female mice cecum samples under different dietary conditions: control diet non-supplemented (NS, orange), supplemented with folic acid (FA, pink) and supplemented with D-*chiro*-inositol (CI, yellow) according to the phylum (**A**–**D**) and the genera (**E**–**G**). (**A**,**E**), Alpha diversity described by the Hill numbers for each group: 0, species richness; 1, modified version of Shannon index; 2, equivalent to Simpson index. Higher number means a stronger focus on dominant taxa. A Mann–Whitney test was applied to alpha diversity values to determine differences among groups of interest. (**B**,**F**) NMDS analysis for beta microbiome diversity. (**C**,**G**) Significant difference (*p* < 0.05) between *Vangl2^+/Lp^* pregnant female mice cecum samples from NS, FA and CI phylum and genus in cecum samples. (**D**) *Firmicutes/Bacteroidetes* ratio.

GraphPad Prism v9 was used to plot and perform the statistical analysis of the functional and morphological data of the intestinal tract. An unpaired two-sided *t*-test was applied to study differences between *Vangl2^+/+^* and *Vangl2^+/Lp^* groups. R v4.3.1 (ggplot2 package) was used to plot the data and perform the statistical analysis of the metagenomics data. The Mann–Whitney test and Permanova were used to study differences among groups (see above). Data were plotted as boxes and whiskers: the median is marked with a line, boxes show the 25−75th percentiles and whiskers show the minimum and maximum (in Figure 2) or 1.5 times the interquartile range (in Figure 3, Figure 4 and Figure 5). *p* < 0.05 was considered statistically significant.

## 3. Results

### 3.1. Gastrointestinal Transit Time, Stool Production and Gut Size in Vangl2^+/+^ and Vangl2^+/Lp^ Mice

NTDs associated with mutations in the *Vangl2* gene have been described as folic acid- and inositol-resistant [29]. This may be either because these nutrients cannot reverse or influence the inherent base defect, or because the intestine is unable to absorb them. To assess whether *Vangl2* mutation affected the gut functionality in *loop-tail* mice, we decided to analyze the gastrointestinal transit time. The transit of food through the intestine is crucial for digestion and absorption of nutrients as well as a key modulator of the host-microbiome interaction. Our results showed that the gastrointestinal transit time of *Vangl2^+/Lp^* dams did not differ to that of *Vangl2^+/+^* mice (*p* = 0.17, *n* = 10 mice per group; Figure 2A). There were no statistically significant differences in the stool production or in the weight and water content of the fecal pellets in *Vangl2^+/Lp^* compared to *Vangl2^+/+^* mice (*p* = 0.78, 0.07 and 0.27, respectively; Figure 2B–D). Having observed no significant changes in the intestinal transit time and stool production, we proceeded to investigate the potential impact of the *Vangl2* mutation on the macroscopic morphology of the gastrointestinal tract. We found that *Vangl2^+/Lp^* mice (*n* = 8) exhibited significantly larger measurements in terms of whole intestine length (*p* = 0.0003), as well as the individual components length (small intestine and colon; *p* = 0.0001 and 0.0005, respectively), along with increased cecum weight (*p* = 0.0039), when compared to the *Vangl2^+/+^* dam mice (*n* = 10; Figure 2E–H).

### 3.2. Analysis of the Microbiota in Relation to the Host Genotype

Genetic, nutritional and gut microbiota-derived factors have been proposed to play a role in gut size [43,44,45], so to further investigate the observed differences in the size of the gastrointestinal system in relation to *Vangl2* genotype, we next explored the composition of the microbiota in the two genotypes of the study: feces of *Vangl2^+/+^* (F-*Vangl2^+/+^*) and feces of *Vangl2^+/Lp^* (F-*Vangl2^+/Lp^*) dams. Alpha and beta diversity analyses did not reveal significant differences at any taxonomical level (Table 1 and Table S1, Figure 3), except at phylum level for Hill number 2 (equivalent to Simpson diversity). However, we did observe a significant decrease in abundance in F-*Vangl2^+/Lp^* for the genera *Prevotella*, *Staphylococcus*, *Faecalibacterium* and *Proteus*. On the other hand, genera *Alistipes*, *Turicibacter*, *Candidatus Arthormitus*, *Anaeroplasma*, as well as two unclassified genera belonging to family *Rikenellaceae* and class *Bacilli* displayed a greater abundance in F-*Vangl2^+/Lp^* (summarized in Table 2 and Figure 3G).

Interestingly, the relative abundance of *Firmicutes* in F-*Vangl2^+/Lp^* females was significantly higher than in their F-*Vangl2^+/+^* counterparts. A parameter to consider related to the health of the individual is the ratio between the phyla *Firmicutes* and *Bacteroidetes* [46]. The relative abundance of *Bacteroidetes* increases as the health conditions of the individual improve, as demonstrated by Turnbaugh et al. [46], in relation to weight loss in obese individuals in both humans and mice. In our research, we observed that among the total analyzed bacteria, the bacterial phyla *Firmicutes* and *Bacteroidetes* exhibited comparable percentages in *Vangl2^+/+^* (43.45 ± 8.24% and 48.68 ± 7.08%, respectively) as well as in *Vangl2^+/Lp^* (49.38 ± 14.38% and 43.48 ± 15.98%, respectively; Table 3), yielding *Firmicutes/Bacteroidetes* ratios of 0.89 and 1.14, respectively. Therefore, this ratio does not appear to vary as a consequence of maternal *Vangl2* genotype (*p* = 0.8; Table 3 and Figure 3D).

### 3.3. Analysis of Microbiota Based on Sample Source

Although the above comparison was made using fresh feces, the cecum represents a unique niche in gastrointestinal microbial ecology that harbors the greatest density and diversity of microbes [47,48]. According to studies by other groups, cecum contents may serve as a more reliable indicator of environmental influences on the gut microbiota compared to fecal samples [49]. The exact reason for this discrepancy is currently unknown, but it appears that the microbial composition within the gastrointestinal tract normalizes during transit through the colon. Therefore, in order to analyze the most appropriate sample, and given the need to sacrifice pregnant dams to study the effect of maternal dietary supplementation on the development of NTDs related to the Wnt/PCP pathway [29], we next compared the microbiota composition between feces (F-*Vangl2^+/Lp^*) and cecum (C-*Vangl2^+/Lp^*) of *Vangl2^+/Lp^* dams.

The comparison between the freshly laid feces and the cecum samples demonstrated a greater difference between these sample types at the genus taxonomical level. In fact, we observed a significant decrease in richness in cecal samples related to feces, while other alpha diversity indices exhibited no differences. Likewise, a beta diversity analysis displays significant changes in microbial composition between cecum and feces of *Vangl2^+/Lp^* dams (Table 1 and Table S1). Differential abundance analysis indicated an increase in feces for the genera *Coprobacillus*, *Lactococcus*, *Enterococcus*, *Candidatus Arthromitus*, *Blautia*, *Escherichia*, *Streptococcus*, *Pseudomonas*, *Lactobacillus*, *Eubacterium*, *Adlercreutzia*, *Staphylococcus* and *Marvinbryantia*, together with other four unclassified genera belonging to the families *Erysipelotrichaceae* and *Barnesiellaceae* and to the classes *Bacilli* and *Betaproteobacteria*. Conversely, we found a decrease in feces for the genera *Helicobacter*, *Dehalobacterium*, *Anaerofustis*, *Mucispirillum*, *Anaerotruncus*, *Bilophila*, *Desulfovibrio* and for an unclassified genus of the family *Lachnospiraceae* (summarized in Table S2). At the phylum taxonomic level, we found an enrichment of *Actinobacteria*, *Bacteroidetes* and *TM7* in the fecal samples, with *Proetobacteria* and *Deferrebacteres* being more enriched in the cecum. It is interesting to note that the relative abundance of *Bacteroidetes* decreased in the cecum compared to the feces, from a *Firmicutes/Bacteroidetes* ratio of 1.14 in the feces to a ratio of 2.00 in the cecum (Table 3).

These results are in line with previous studies that reported a different composition of the microbiota found in feces and the cecum in mouse [49], humans [47], chickens [50] and pigs [48]. Given that the cecum appears to be the main anatomical site where environmental influences were reflected in the composition of the microbiota [51], we decided to analyze cecum contents for our following studies on the impact of maternal diet on NTDs occurrence and the gut microbiota.

### 3.4. Analysis of Microbiota According to Gestation

We next tested whether pregnancy might alter the gut microbiota, for which cecum samples were compared from non-pregnant and 12.5 days pregnant *Vangl2^+/Lp^* mice (C-*Vangl2^+/Lp^* and C-*Vangl2^+/Lp^*-P, respectively). The day of pregnancy chosen was in accordance with the date of sacrifice of the females for embryo extraction and evaluation of presence and severity of NTDs [29]. Diversity analysis showed no clear changes between C-*Vangl2^+/Lp^* and C-*Vangl2^+/Lp^*-P samples (Table 1 and Table S1, Figure 4). Nevertheless, our findings revealed a significant decrease in the prevalence of *Turicibacter* and *Anaerofustis* and a concomitant significant increase in *Faecalibacterium* among C-*Vangl2^+/Lp^*-P samples. Despite *Turicibacter*, *Anaerofustis* and *Faecalibacterium* being part of the *Firmicutes* phylum, our observations revealed no notable variances in taxa abundance at the phylum level between the C-*Vangl2^+/Lp^* and C-*Vangl2^+/Lp^*-P samples (59.36 ± 10.50% and 63.91 ± 9.72%, respectively; Table 1, Table S1 and Table 4, and Figure 4). However, in this context, our observations showed a decrease in the relative abundance of *Bacteroidetes* during the non-pregnant state compared to the pregnant state (29.61 ± 10.59% and 23.51 ± 8.02%, respectively). This resulted in a shift, although it was not statistically significant, in the *Firmicutes/Bacteroidetes* ratio from 2.00 in C-*Vangl2^+/Lp^* individuals to 2.72 in C-*Vangl2^+/Lp^*-P samples (Table 3 and Figure 4C).

### 3.5. Analysis of Microbiota in Response to Dietary Supplementation

To investigate the effect of dietary supplementation on gut microbiota composition, cecum samples were analyzed from *Vangl2^+/Lp^* pregnant dams at E12.5 on three different diets: non-supplemented control diet (C-*Vangl2^+/Lp^*-P-NS, same samples as in C-*Vangl2^+/Lp^*-P), folic acid supplementation (C-*Vangl2^+/Lp^*-P-FA) and *D-chiro*-inositol supplementation (C-*Vangl2^+/Lp^*-P-CI).

The study of gut microbiota following FA supplementation showed that in the C-*Vangl2^+/Lp^*-P-FA group, the alpha and beta diversity analyses suggest negligible differences when compared to C-*Vangl2^+/Lp^*-P-NS (Table 1 and Table S1, Figure 5). Only at phylum level could we observe barely significant changes in richness between these two groups. However, the genera *Anaerofustis*, *Marvinbryantia* and *Bilophila* manifested an increase under FA supplementation, while *Butyricicoccus*, *Faecalibacterium* and *Sutterella* had greater abundances in the C-*Vangl2^+/Lp^*-P-NS. In our analysis, we observed that the C-*Vangl2^+/Lp^*-P-NS samples comprised 63.91% *Firmicutes* and 23.51% *Bacteroidetes*, whereas the C-*Vangl2^+/Lp^*-P-FA samples contained 70.18% *Firmicutes* and 16.09% *Bacteroidetes*. These findings resulted in *Firmicutes/Bacteroidetes* ratios of 2.72 and 4.36, respectively, with a nearly statistically significant difference (*p* = 0.05; summarized in Table 3 and Table 5A, and Figure 5).

In regards to CI supplementation, the group C-*Vangl2^+/Lp^*-P-CI showed a marginally significant increase in richness related to C-*Vangl2^+/Lp^*-P-NS across several taxonomic levels. However, beta diversity analysis revealed no clear changes in community structure between these groups. Within phyla, the relative abundance of *Deferribacteres* in C-*Vangl2^+/Lp^*-P-CI females was significantly lower than in their C-*Vangl2^+/Lp^*-P-NS counterparts. Interestingly, C-*Vangl2^+/Lp^*-P-CI group presented a drastic decrease in the abundance of the genus *Mucispirillium*, the only genus in the *Deferribacterota* phylum present in the samples. Genera *Paraprevotella* (phylum *Bacteroidetes*) and *Desulfomicrobium* (phylum *Thermodesulfobacteriota*) were also significantly lower in this supplemented group when compared to the C-*Vangl2^+/Lp^*-P-NS. On the other hand, from the phyla *Firmicutes* we found a significant increase for the genera *Enterococcus*, *Turicibacter*, *Anaerofustis*, *Coprococcus*, *Marvinbryantia*, *Nitrosomonas* and two additional unclassified genera from the family *Mogibacteriaceae* and class *Bacilli* in the C-*Vangl2^+/Lp^*-P-CI group. In addition, and from the *Philum Proteobacteria*, the genera *Nitrosomonas* was also increased in the gut microbiota from C-*Vangl2^+/Lp^*-P-CI dams. In this context, C-*Vangl2^+/Lp^*-P-CI samples contained 68.66% *Firmicutes* and 22.35% *Bacteroidetes*, figures that did not significantly alter the *Firmicutes/Bacteroidetes* ratio compared to C-*Vangl2^+/Lp^*-P-NS (summarized in Table 3 and Table 5B, and Figure 5).

## 4. Discussion

In the present study, we used the *loop-tail* mouse model for NTDs that exhibits 100% penetrance of craniorachischisis in homozygotes [52] and both open and closed caudal NTDs in heterozygotes [26,29]. An interesting feature of the phenotype of these mutants is that they are resistant to folic acid and inositol supplementation [28,29]. This observation implies that the morphological changes induced in the embryo by the *Vangl2* mutation cannot be rectified by an increase in folic acid and/or inositol levels during the critical neurulation process. A plausible explanation for this could be that the *Vangl2* mutation is altering the development of the intestine, as described in *Xenopus* [30], consequently affecting the proper absorption of essential nutrients. In our experiments, we found that while gastrointestinal transit time and stool production remained consistent between *Vangl2^+/Lp^* and *Vangl2^+/+^* mice, there was a noticeable variation in intestinal length and cecal weight based on the *Vangl2* genotype, as well as in certain microbiota composition (Figure 2 and Figure 3). A previous study exploring the impact of host genetics on the composition of the gut microbiota revealed a connection between the abundance of *Sutterellacea* and a single nucleotide polymorphism (SNP) in the *VANGL1* gene, which is closely related to *VANGL2* and highly expressed in gut tissues [53]. These findings may suggest that morphological changes in the gut produced by the *Vangl2* mutation may have an impact on the composition of the microbiota and perhaps on nutrient absorption. Further work along these lines will clarify this interesting issue.

NTDs have been proposed as multifactorial diseases for which both obesity and diabetes mellitus are considered risk factors. We have previously shown that embryopathies associated with mutants of the Wnt/PCP pathway were aggravated in combination with maternal diabetes [54]. In the present study, we found that *Vangl2* mutation caused dysbiosis (Figure 3) that was in line with microbiota alterations associated with obesity and gestational diabetes mellitus. At the phylum level, *Vangl2^+/Lp^* dams showed an increased abundance of *Firmicutes* compared to their *Vangl2^+/+^* counterpart, an alteration previously observed in patients with obesity and in gestational diabetes mellitus [55,56]. However, although a higher *Firmicutes/Bacteroidetes* ratio has been linked to obesity [46], in our study, the increase in *Firmicutes* in *Vangl2^+/Lp^* did not translate into an altered *Firmicutes/Bacteroidetes* ratio (Table 3). At the genus level, we observed an increase in the *f_Rikenellaceae_unclassified* and *Alistipes* genera in *Vangl2^+/Lp^* females with respect to *Vangl2^+/+^* (Table 2). Increased abundance of an unassigned genera of *Rikenellaceae* and *Alistipes* has been previously associated with gestational diabetes mellitus [56,57,58]. Following the results presented here, future research could consider the possibility that the microbiota of the *Vangl2^+/Lp^* females, which resembles microbiota alteration observed in gestational diabetes mellitus and obesity, could be playing a determinant role in the phenotype of this NTD model.

In addition to our comparative metagenomics analysis by genotype, we also examined the microbiota composition of feces and cecum of *Vangl2^+/Lp^* mice. We observed substantial differences between cecal and fecal samples (Table S2), as has been shown in different vertebrates [47,48,49,50]. The cecum is an area of the gut where fermentation of dietary fiber occurs and is home to a diverse microbial community [59]. The difference in abundance of certain microbial populations relative to other parts of the gut may be due to the ability of a particular population to thrive in this fermentative environment. This may explain why we observed an increase in *Proteobacteria* and *Deferribacteria* phyla in the ceca. On the other hand, feces are composed of undigested food particles, sloughed cells and microbial populations that have moved through the gastrointestinal tract. As microbial communities move through the gut, some bacterial groups may become more enriched in feces due to their ability to resist and survive. This may be the reason why we detected higher numbers of *Saccharibacteria (TM7)*, *Bacteroidetes* and *Actinobacteria* in feces compared to cecal samples.

Another determining factor in our study was the pregnancy status of mice. Changes in hormone secretion and immune responses during pregnancy are commonly known as maternal metabolic disturbance syndrome, which is characterized by insulin resistance [60], inflammation [61] and oxidative stress [62]. This condition is often accompanied by an increase in mucosa-degrading microbiota [63,64] and intestinal permeability [65], affecting the gut microbiota richness and likely altering the maternal metabolome [66]. Although this transformation develops gradually, the most notable divergence is observed during the later stages of pregnancy [67,68]. Our cecal samples of *Vangl2^+/Lp^* pregnant dams were collected at mid-gestation (E12.5) when we found no major differences at the phylum level compared with the non-pregnant counterpart dams, but few changes at the genus taxonomic level (Figure 4 and Table 4). In line with previous research findings [69,70], we found an enrichment of *Faecalibacterium (Firmicutes* phylum) in pregnant *Vangl2^+/Lp^* dams, a butyrate-producing bacterium known for its anti-inflammatory effects. Other groups have suggested that the augmented abundance of *Faecalibacterium* during pregnancy serves as a corrective mechanism to counteract the low-grade inflammation that may occur during pregnancy, which is a potentially detrimental condition to the fetus [70]. In addition, our research sheds light on the relatively understudied *Turicibacter* and *Anaerofustis* genus from the *Firmicutes* phylum, emphasizing its susceptibility to gestational status, as highlighted by a recent study of sow microbiota [71]. In our study, *Turicibacter* was found to be significantly decreased during pregnancy, which is in line with results from previous publications [72]. *Anaerofustis*, characterized as a beneficial bacterium, promotes fiber digestion and the production of short-chain fatty acids, which could enhance intestinal antioxidant properties and morphological structures in animals [73]. Therefore, a reduction in the abundance of *Anaerofustis* and *Turicibacter* could be related to the metabolic alterations that accompany the maternal metabolic disturbance syndrome and their possible implication on the richness of the gut microbiota.

Folate plays a crucial role in various cellular processes essential for fetal development, serving as cofactor in one-carbon transfer reactions important for DNA and RNA synthesis, DNA repair, and amino acid metabolism [74]. Folate, essential for preventing NTDs during early pregnancy, cannot be naturally produced by mammalian cells. As a result, its availability primarily relies on dietary intake and supplementation, making it highly recommended for women to ensure adequate levels before and during pregnancy [75,76]. Interestingly, certain bacteria residing in the gut have been shown to possess the ability to synthesize folate and other B vitamins, as indicated by the presence of folate biosynthesis pathways [77,78,79]. Nearly all *Bacteroidetes*, *Fusobacteria* and *Proteobacteria* in the human gut contain all the functional components for de novo folate synthesis, while this is rare in *Actinobacteria* and *Firmicutes* [80]. It has been estimated that the collective folate production by these gut bacteria could account for up to 37% of the Dietary Reference Intake [80]. Our results showed that *Vangl2^+/Lp^* had a significant increase in comparison to *Vangl2^+/+^* samples in the composition of two genera belonging to the *Bacteriodetes* phylum, *Alistipes* and an unclassified genus of the *Rikenellaceae* family (Table 2), as well as an elevation of *Lactobacillales_unclassified*, one of the folic acid-producing order of the phylum *Firmicutes* [81]. These findings indicate an increase in the folic acid-producing populations associated with the *Vangl2* mutant genotype, which may be due to a potential demand for folate. More studies are needed to determine whether, as in *Xenopus* [30], Vangl2 in mammals plays an important role in the formation of the digestive tract with a relevant effect on folate absorption.

The importance of folate goes beyond its impact on the host and on fetal development, as the gut microbiota also uses this vitamin for its own growth and composition [82]. In this study, we have observed that folic acid (FA) supplementation reduces the relative abundance of *Sutterella*, (Table 5A), a *Proteobacteria* with a known pro-inflammatory capacity that is positively associated with gestational diabetes mellitus and obesity [58]. Furthermore, *Sutterella* has been linked to an increased capacity for folate production in humans [83]. This finding raises the possibility that when individuals receive FA supplementation, there may be a reduced need for microbiota-driven folate production to counterbalance the surplus of circulating folate. We also observed that FA supplementation increased the abundance of *Anaerophustis* (Table 5A), a genus of *Firmicutes* phylum described as beneficial bacteria [73]. These results suggest that FA supplementation may not only contribute to supporting the growth of beneficial bacteria, but also help to reduce pro-inflammatory activity during pregnancy. These findings pave the way towards a deeper understanding of how FA could positively influence both maternal health and the gut microbiome, promoting overall wellbeing during pregnancy.

A previous study of folate-producing probiotics in human microbiota cultures showed that these probiotics led to an increase in the *Faecalibacterium* and *Butyricicoccus* genera [84]. However, our in vivo mouse model results, which are consistent with findings from an in vivo chicken model [85], revealed a negative correlation between the relative abundance of *Faecalibacterium* and *Butyricicoccus* and FA supplementation (Table 5A). These contrasting outcomes between the in vitro and in vivo experiments underscore the complex and context-dependent nature of the interactions between folate-producing probiotics and FA supplementation. Furthermore, it is worth highlighting the elevated *Firmicutes/Bacteroidetes* ratio observed in response to FA supplementation when compared to a non-supplemented diet (Table 3). This increased ratio has been linked to obesity [46], which in turn raises the risk of NTDs. Intriguingly, studies involving various NTD mouse models have shown that FA supplementation can have adverse effects on neurulation and embryonic survival [86]. These findings suggest the possibility that alterations in microbiota composition due to FA supplementation may also contribute to the development of NTDs under certain genetic conditions. All these observations highlight the need for further research to decipher the underlying mechanisms and to consider the influence of host factors in shaping the gut microbiota response to FA interventions. Such investigations could provide valuable insights into optimizing the potential benefits of FA supplementation on gut microbial populations in different biological settings.

Inositol is a natural six-carbon sugar alcohol compound involved in many biological pathways; it is often referred to as a pseudo-vitamin (vitamin Bh or B8). Inositol can be synthesized from glucose [87] and is also present in various forms in the human diet, including its free form, inositol-containing phospholipids, and as phytic acid (inositol polyphosphate or InsP6), the latter being the most abundant form in foods of plant origin [88]. A significant portion of dietary InsP6 is degraded during digestion, especially in the large intestine, where bacterial phytases and phosphatases break down InsP6, releasing free inositol and other inositol phosphate derivatives [89,90]. Dietary InsP6 can increase the capacity of intestinal microbiota to degrade InsP6, leading to increased inositol production and absorption [91].

Here, we have shown that D-*chiro*-inositol (CI) supplementation of *Vangl2^+/Lp^* females had a significant impact on the cecal microbiota at the phylum level (Figure 5). There was a clear decrease in *Deferribacteres*, including the genus *Mucispirillum*, even the species *Schaedleri* (Table 5B). *Mucispirillum* is to date the only genus of the *Deferribacteraceae* known to inhabit the gastrointestinal tract of vertebrates [92]. Previous research has linked an increase in *Mucispirillum* to high-fat diets, drug treatment, stress and certain diseases such as Parkinson’s disease, rheumatoid disease or arthritis [92]. Moreover, *M. schaedleri* can breathe nitrate [93], which becomes abundant in inflammatory conditions, possibly explaining its association with inflammation. Therefore, the reduction in the relative abundance of *M. schaedleri* observed in our study may indicate a healthier gut environment induced by CI supplementation. No dysbiosis was detected in the *Firmicutes* population at the taxonomic phylum level. However, there was an increase in the relative abundance of seven genera within this phylum: *c_Bacilli_unclassified*, *Enterococcus*, *Turicibacter*, *Anaerofustis*, *f_Mogibacteriaceae_unclassified*, *g_Coprococcus* and *Marvinbryantia* (Table 5B). These genera have previously been negatively associated with gestational diabetes mellitus: *c_Bacilli_unclassified* (as a genus of *Bacilli* class) [94]; *Enterococcus* [95]; *Marvinbryantia* [96]; and *Anaerofustis*, *f_Mogibacteriaceae_unclassified*, *g_Coprococcus* and *Marvinbryantia*, as genus from the *Clostridiales* class [97]. Diabetes and hyperglycemia during pregnancy are known risk factors for the development of NTDs [98], and both can also affect the patient’s microbiota [99]. Inositol and its derivatives have shown beneficial health effects, such as being antioxidants, anti-inflammatory, anti-cancer and anti-diabetic agents [87]. In the context of our experimental model, inositol supplementation has been found to counteract the teratogenic effects of diabetes and hyperglycemia on NTDs both in vitro and in vivo [100,101,102]. Our study highlights how dietary inositol supplementation may influence specific microbial populations, potentially contributing to mitigating the deleterious effects of diabetes on maternal and fetal health.

## 5. Conclusions

In the present study, we observed morphological changes in the intestinal tract of *Vangl2^+/Lp^* mice, likely resulting from developmental alterations driven by the *Vangl2* mutation. *Vangl2^+/Lp^* mice also showed alterations in their microbiota, with elevated populations capable of producing folic acid, which suggests a potential increased demand for this nutrient, either by the host or the microbiota. Additionally, the dysbiosis observed in *Vangl2* mutants resembles the dysbiosis seen in situations of obesity and gestational diabetes mellitus, both of which are risk factors for NTDs. This research may contribute to opening a new avenue for understanding the complex relationship between the microbiota, the genotype and the development of NTDs.

## Figures and Tables

**Table 1:** Alpha and beta diversity analyses at the phylum (**A**) and genus (**B**) taxonomical levels.

Comparison	Alpha			Beta
	0	1	2	
(A) Phylum level				
F-*Vangl2^+/+^* vs. F-*Vangl2^+/Lp^*	0.17	0.26	0.05	0.80
F-*Vangl2^+/Lp^* vs. C-*Vangl2^+/Lp^*	0.37	0.14	0.73	0.27
C-*Vangl2^+/Lp^* vs. C-*Vangl2^+/Lp^*-P	0.37	0.86	0.67	0.80
C-*Vangl2^+/Lp^*-P-NS vs. C-*Vangl2^+/Lp^*-P-FA	0.08	0.44	0.16	0.27
C-*Vangl2^+/Lp^*-P-NS vs. C-*Vangl2^+/Lp^*-P-CI	N/A	0.09	0.26	0.27
(B) Genus level				
F-*Vangl2^+/+^* vs. F-*Vangl2^+/Lp^*	0.69	0.16	0.14	0.34
F-*Vangl2^+/Lp^* vs. C-*Vangl2^+/Lp^*	0.005	0.30	0.67	0.01
C-*Vangl2^+/Lp^* vs. C-*Vangl2^+/Lp^*-P	0.14	0.14	0.49	0.65
C-*Vangl2^+/Lp^*-P-NS vs. C-*Vangl2^+/Lp^*-P-FA	0.20	1	1	0.34
C-*Vangl2^+/Lp^*-P-NS vs. C-*Vangl2^+/Lp^*-P-CI	0.06	0.22	0.39	0.34

Alpha diversity is described by the Hill’s Diversity (qD) for each group, and for different numbers (q): 0 (equals to species richness), 1 (modified version of Shannon index) and 2 (equivalent to Simpson index). F, feces; C, cecum; P, pregnant; NS, non-supplemented; FA, supplemented with folic acid; CI, supplemented with D-*chiro*-inositol; N/A, data not available. *n* = 9 female mice per group.

**Table 2:** Significant differences at the genus level according to the host genotype in feces from *Vangl2^+/+^* and *Vangl2^+/Lp^* female mice (*n* = 9 per group).

Phylum	Genus	logFC	*p*-Value	FDR
*p_Bacteroidetes*	*g_Prevotella*	2.39	6.20 × 10^−3^	4.20 × 10^−2^
	*f_Rikenellaceae_unclassified*	−1.72	0	1.00 × 10^−4^
	*g_Alistipes*	−1.15	1.90 × 10^−3^	2.50 × 10^−2^
*p_Firmicutes*	*c_Bacilli_unclassified*	−0.97	1.20 × 10^−3^	2.41 × 10^−2^
	*g_Staphylococcus*	2.61	0	1.00 × 10^−4^
	*o_Lactobacillales_unclassified*	−1.40	2.20 × 10^−3^	2.50 × 10^−2^
	*g_Turicibacter*	−4.96	2.00 × 10^−4^	4.10 × 10^−3^
	*g_Candidatus_Arthromitus*	−2.42	2.50 × 10^−3^	2.50 × 10^−2^
	*g_Faecalibacterium*	1.26	2.10 × 10^−3^	2.50 × 10^−2^
*p_Proteobacteria*	*g_Proteus*	1.03	4.70 × 10^−3^	3.48 × 10^−2^
*p_Tenericutes*	*g_Anaeroplasma*	−5.19	3.70 × 10^−3^	3.31 × 10^−2^

The significant changes were determined using a Zero-inflated Gaussian mixture model using metagenomeSeq. logFC, log2 foldchange; FDR, False Discovery Rate adjusted *p*-value.

**Table 3:** *Firmicutes/Bacteroidetes* ratio.

Groups	% *Firmicutes*	% *Bacteroidetes*	*Firmicutes/Bacteroidetes*
F-*Vangl2^+/+^*	43.45 ± 8.24	48.68 ± 7.08	0.89
F-*Vangl2^+/Lp^*	49.38 ± 14.38	43.48 ± 15.98	1.14
C-*Vangl2^+/Lp^*	59.36 ± 10.50	29.61 ± 10.59	2.00
C-*Vangl2^+/Lp^*-P-NS	63.91 ± 9.72	23.51 ± 8.02	2.72
C-*Vangl2^+/Lp^*-P-FA	70.18 ± 4.84	16.09 ± 5.44	4.36
C-*Vangl2^+/Lp^*-P-CI	68.66 ± 8.98	22.35 ± 8.94	3.07

This table displays the mean percentage ± SD of *Firmicutes* and *Bacteroidetes* within the overall microbiota phylum from each of the study groups and the value of the *Firmicutes/Bacteroidetes* ratio. F, feces; C, cecum; P, pregnant; NS, non-supplemented; FA, supplemented with folic acid; CI, supplemented with D-*chiro*-inositol. *n* = 9 female mice per group.

**Table 4:** Significant differences at the genus level in cecal samples of pregnant and non-pregnant *Vangl2^+/Lp^* female mice (*n* = 9 per group).

Phylum	Genus	logFC	*p*-Value	FDR
*p_Firmicutes*	*g_Turicibacter*	−6.77	1.62 × 10^−5^	6.56 × 10^−4^
	*g_Anaerofustis*	−3.62	3.24 × 10^−6^	2.62 × 10^−4^
	*g_Faecalibacterium*	1.01	9.92 × 10^−4^	2.68 × 10^−2^

The significant changes were determined using a Zero-inflated Gaussian mixture model using metagenomeSeq. logFC, log2 foldchange; FDR, False Discovery Rate adjusted *p*-value.

**Table 5:** Significant differences at the genus level observed based on the supplementation of the diet level in cecal samples of pregnant *Vangl2^+/Lp^* mice (*n* = 9 per group). (**A**) Significant changes in non-supplemented vs FA-supplemented mice. (**B**) Significant changes in non-supplemented vs CI-supplemented mice.

Phylum	Genus	logFC	*p*-Value	FDR
(A) FA-supplemented mice				
*p_Firmicutes*	*g_Anaerofustis*	−1.21	1.40 × 10^−3^	1.88 × 10^−2^
	*g_Marvinbryantia*	−1.17	9.65 × 10^−5^	2.61 × 10^−3^
	*g_Butyricicoccus*	2.15	2.69 × 10^−6^	1.09 × 10^−4^
	*g_Faecalibacterium*	1.02	6.68 × 10^−4^	1.08 × 10^−2^
*p_Proteobacteria*	*g_Sutterella*	5.46	2.55 × 10^−9^	2.06 × 10^−7^
	*g_Bilophila*	−2.08	2.94 × 10^−3^	3.40 × 10^−2^
(B) CI-supplemented mice				
*p_Bacteroidetes*	*g_Paraprevotella*	3.00	4.36 × 10^−3^	3.21 × 10^−2^
*p_Deferribacteres*	*g_Mucispirillum*	3.57	1.70 × 10^−5^	6.88 × 10^−4^
*p_Firmicutes*	*c_Bacilli_unclassified*	−1.19	1.68 × 10^−4^	3.40 × 10^−3^
	*g_Enterococcus*	−2.63	1.43 × 10^−3^	1.65 × 10^−2^
	*g_Turicibacter*	−4.64	1.52 × 10^−4^	3.40 × 10^−3^
	*f_[Mogibacteriaceae]_unclassified*	−1.02	2.56 × 10^−3^	2.22 × 10^−2^
	*g_Anaerofustis*	−1.21	5.00 × 10^−4^	8.09 × 10^−3^
	*g_Coprococcus*	−1.33	2.74 × 10^−3^	2.22 × 10^−2^
	*g_Marvinbryantia*	−1.02	2.58 × 10^−3^	2.22 × 10^−2^
*p_Proteobacteria*	*g_Nitrosomonas*	−1.97	4.84 × 10^−6^	3.92 × 10^−4^
	*g_Desulfomicrobium*	2.95	1.29 × 10^−3^	1.65 × 10^−2^

The significant changes were determined using a Zero-inflated Gaussian mixture model using metagenomeSeq. logFC, log2 foldchange; FDR, False Discovery Rate adjusted *p*-value.

## Data Availability

The data for this study have been deposited in the European Nucleotide Archive (ENA) at EMBL-EBI under accession number PRJEB68194 (https://www.ebi.ac.uk/ena/browser/view/PRJEB68194; accessed on 4 November 2023).

## Supplementary Materials

The following supporting information can be downloaded at: https://www.mdpi.com/article/10.3390/nu15234944/s1, Table S1: Study of global differences in microbial composition among experimental groups using a permutational ANOVA (permanova); Table S2: Significant differences at the genus level of microbiota from feces and cecum of non-pregnant *Vangl2^+/Lp^* female mice.
