# Supplementary material for: Analysis of Gut Characteristics and Microbiota Changes with Maternal Supplementation in a Neural Tube Defect Mouse Model
Source: Nutrients. 2023 Nov 28;15(23):4944. doi: 10.3390/nu15234944 (PMC10708240; doi:10.3390/nu15234944)
Supplement: Supplementary file 1 [file nutrients-15-04944-s001.zip › nutrients-2692791-supplementary.pdf]

Supplementary Materials:

Table S1. Study of global differences in microbial composition among experimental groups using a permutational ANOVA (permanova). (A) Permanova results for beta diversitity at phylum level. (B) Permanova results for beta diversitity at genus level.

Comparisons	Df	SumsOfSqs	F.Model	R2	*p*-value	*p*-adjusted
**(A) Phylum beta diversity permanova**						
F-*Vangl2*$^{+/+}$ vs F-*Vangl2*$^{+/Lp}$	1	0.029	0.941	0.056	0.33	0.801
F-*Vangl2*$^{+/Lp}$ vs C-*Vangl2*$^{+/Lp}$	1	0.138	3.875	0.195	0.067	0.268
C-*Vangl2*$^{+/Lp}$ vs C-*Vangl2*$^{+/Lp}$-P	1	0.027	1.315	0.076	0.276	0.801
C-*Vangl2*$^{+/Lp}$-P-NS vs C-*Vangl2*$^{+/Lp}$-P-FA	1	0.044	3.756	0.190	0.053	0.265
C-*Vangl2*$^{+/Lp}$-P-NS vs C-*Vangl2*$^{+/Lp}$-P-CI	1	1	0.020	1.176	0.068	0.267
**(B) Genus beta diversity permanova**						
F-*Vangl2*$^{+/+}$ vs F-*Vangl2*$^{+/Lp}$	1	0.058	2.211	0.121	0.084	0.336
F-*Vangl2*$^{+/Lp}$ vs C-*Vangl2*$^{+/Lp}$	1	0.153	5.815	0.267	0.001	0.005
C-*Vangl2*$^{+/Lp}$ vs C-*Vangl2*$^{+/Lp}$-P	1	0.012	0.629	0.038	0.647	0.647
C-*Vangl2*$^{+/Lp}$-P-NS vs C-*Vangl2*$^{+/Lp}$-P-FA	1	0.026	1.661	0.094	0.152	0.336
C-*Vangl2*$^{+/Lp}$-P-NS vs C-*Vangl2*$^{+/Lp}$-P-CI	1	0.033	1.898	0.106	0.111	0.336

F, feces; C, cecum; P, pregnant; NS, non-supplemented; FA, supplemented with folic acid; CI, supplemented with D-*chiro*-inositol. n = 9 female mice per group.

Table S2. Significant differences at the genus level of microbiota from feces and cecum of non-pregnant *Vangl2*$^{+/Lp}$ female mice (*n* = 9 per group).

Phylum	Genus	logFC	*p*-value	FDR
p_Actinobacteria	*g_Adlercreutzia*	-1.57	8.98×10^{-3}	3.46×10^{-2}
p_Bacteroidetes	*f_[Barnesiellaceae]_unclassified*	-0.85	1.41×10^{-3}	8.14×10^{-3}
p_Deferribacteres	*g_Mucispirillum*	2.71	6.88×10^{-4}	5.94×10^{-3}
p_Firmicutes	*c_Bacilli_unclassified*	-1.24	2.84×10^{-4}	3.28×10^{-3}
	g_Staphylococcus	-0.74	1.00×10^{-2}	3.69×10^{-2}
	g_Enterococcus	-3.05	2.74×10^{-4}	3.28×10^{-3}
	g_Lactobacillus	-1.79	2.45×10^{-3}	1.17×10^{-2}
	g_Lactococcus	-2.78	2.40×10^{-4}	3.28×10^{-3}
	g_Streptococcus	-1.52	1.32×10^{-3}	8.14×10^{-3}
	g_Candidatus_Arthromitus	-2.93	5.84×10^{-4}	5.91×10^{-3}
	g_Dehalobacterium	1.43	2.79×10^{-5}	1.13×10^{-3}
	g_Anaerofustis	2.55	1.35×10^{-4}	3.28×10^{-3}
	f_Lachnospiraceae_unclassified	0.62	7.86×10^{-3}	3.18×10^{-2}
	g_Blautia	-1.01	7.33×10^{-4}	5.94×10^{-3}
	g_Marvinbryantia	-0.98	1.38×10^{-2}	4.46×10^{-2}
	g_Anaerotruncus	2.38	9.23×10^{-4}	6.27×10^{-3}
	f_Erysipelotrichaceae_unclassified	-1.85	1.43×10^{-2}	4.46×10^{-2}
	g_[Eubacterium]	-1.37	5.66×10^{-3}	2.41×10^{-2}
	g_Coprobacillus	-2.71	2.15×10^{-4}	3.28×10^{-3}
p_Proteobacteria	*c_Betaproteobacteria_unclassified*	-0.97	1.36×10^{-2}	4.46×10^{-2}
	g_Bilophila	2.12	2.16×10^{-3}	1.10×10^{-2}
	g_Desulfovibrio	1.60	1.16×10^{-2}	4.10×10^{-2}
	g_Helicobacter	3.06	0	1.30×10^{-6}
	g_Escherichia	-1.37	9.29×10^{-4}	6.27×10^{-3}

The significant changes were determined using a Zero-inflated Gaussian mixture model using metagenomeSeq. logFC, log2 foldchange; FDR, False Discovery Rate adjusted *p*-value.
